# INTUITION: a data platform to integrate human epilepsy clinical care and support for discovery

**DOI:** 10.3389/fdgth.2023.1091508

**Published:** 2023-06-08

**Authors:** Biswajit Maharathi, Fozia Mir, Karthik Hosur, Jeffrey A. Loeb

**Affiliations:** ^1^ Department of Neurology and Rehabilitation, University of Illinois, Chicago, IL, United States; ^2^ Center for Clinical and Translational Science, University of Illinois at Chicago, Chicago, IL, United States

**Keywords:** epilepsy, systems biology, integrated informatics, neurology, intuition

## Abstract

To make appropriate clinical decisions, clinicians consider many types of data from multiple sources to arrive at a diagnosis and plan. However, the current health systems have siloed data, making it challenging to develop information platforms that integrate this process into a single place for comprehensive clinical evaluation and research. INTUITION is a human brain integrative data system that facilitates multimodal data integration, unified storage, cohort selection, and analysis of multidisciplinary datasets. In this article, we describe the use of INTUITION to include electronic health records together with co-registered neuroimaging and EEG from patients who undergo invasive brain surgery for epilepsy. In addition to providing clinically useful visualizations and analytics to help guide surgical planning, INTUITION also links a bank of human brain epileptic tissues from specific brain locations to quantitative EEG, imaging, histology, and omics studies in a unique, completely integrated informatics platform. Having a clinically useful platform for integrating multimodal datasets can not only aid in clinical management decisions but also in creating a unique resource for research and discovery when linked to spatially mapped tissue samples.

## Introduction

### Epilepsy: a challenging case with enormous potential

Approximately one-third of all epilepsy patients are resistant to anti-epileptic drugs (1). Some of these patients benefit from surgical resection of the epileptic brain tissue to become seizure-free. The decision to surgically resect brain tissue requires accurate localization of seizure onset. The tissue localization process requires integrated evaluation of multimodal data derived from Electronic Health Record (EHR), spatially co-registered electrodes to the brain surface, evaluation of intracranial Electroencephalographs (EEGs), imaging, neuropsychiatric tests, and lab testing. The tissue is then removed following detailed group meeting discussions to assess outcome (seizure freedom or reduction). Tissue that is precisely mapped to the underlying electrical signals offers a unique opportunity to explore the causes of epilepsy and develop new treatments (2). Until recently, major limitations in maximizing the research utility of tissue removed from epilepsy surgery patients have been finding ways to link different data modalities, establishing streamlined data processing pipelines, and enabling integrated informatics. The situation is compounded since multidisciplinary data types needed for clinical care and interdisciplinary research are siloed on different computer systems. This requires data aggregation, curation, quality control, inventory management for different data coming from various sources through different data collection protocols, and robust governance that takes care of security, compliance, and data access based on the specific needs of researchers.

The removal of human brain tissue to treat or cure epileptic disorders offers an exceptional research opportunity that is not possible in most other human brain disorders. Critical to the success of this research is precise co-registration of tissue with both the location of intracranial recording electrodes and multimodal imaging as described in a recent review (2). This has allowed us to study the cellular (histological) and omics (genomic, proteomic, metabolomics) correlates of specific physiological and anatomical measures from EEGs and imaging studies of multiple epileptic brain regions. Without this localization, tissue removed has limited value since there is significant heterogeneity in the electrical signals in different brain regions determined from intracranial recordings. The integrated understanding of electrophysiology, neuroimaging, histology, omics information, along with patient history, can elucidate the complex mechanism of epilepsy and significantly advance the field.

### Creating a multidimensional database of the human epileptic brain

Several investigator-led initiatives have created comprehensive neurological disease-related databases for the past few decades to enhance research and knowledge discovery. Such databases store large, curated datasets, including EEG, imaging, genomics, and clinical details. Epilepsy is a common neurological condition of recurrent seizures, where many diverse types of data are used to evaluate and surgically treat patients who fail to respond to medical management. Platforms such as IEEG.ORG, EPILEPSIAE (3), and Temple EEG Database (4) store EEG datasets along with clinical and imaging metadata specifically for epilepsy. There are also databases that store more specific EEG datasets like the neonatal EEG database (5). On the other hand, databases like EpimiRBase (6) store epilepsy related microRNA datasets, and several imaging databases store imaging modality specific high resolution brain scans for humans (7) and animals along with genomic information (8). There are additional disease and condition specific initiatives such as LONI (9) which also hold both EEG and imaging information along with clinical details for traumatic brain injury and epilepsy, and our own PTRD database (10) that holds clinical and preclinical data on subarachnoid hemorrhage that includes electronic health records, imaging, EEG, and derived research information along with intuitive visualizations on patients and animal models.

**Figure 1:**
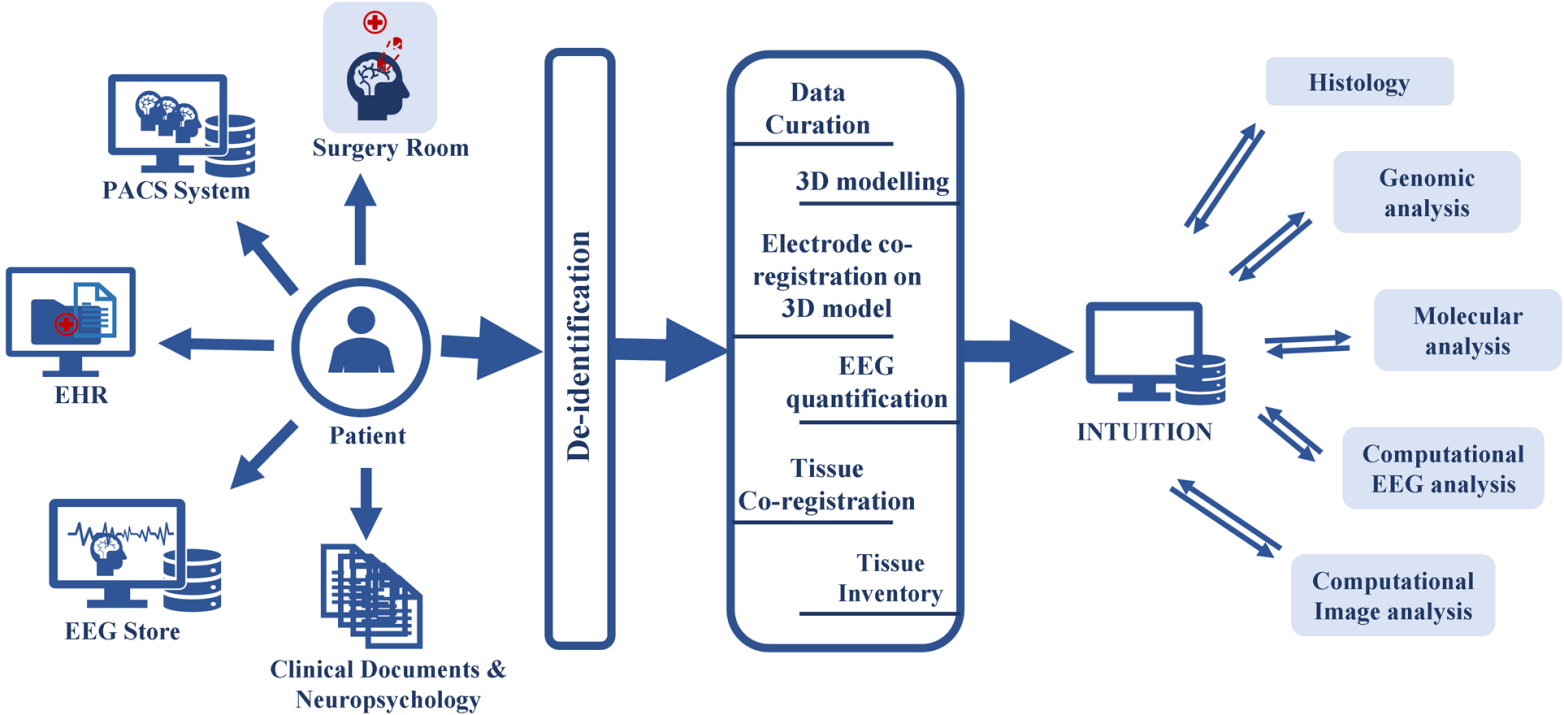
INTUITION workflow depicting upstream and downstream data flow. INTUITION has two distinct data flows. In the upstream data flow, INTUITION receives patient demographic information (EHR), brain imaging (PACS system), EEG and other electrophysiology data (EEG Store), tissue samples, intraoperative photographs (surgery room), other clinical notes, reports, neuropsychology notes, surgical evaluations, and physician observations (clinical documents not covered anywhere else). It then de-identifies the information and passes to an intermediate stage, where the data is curated, and tissue samples are co-registered, tagged, and updated in the inventory. All verified data is then entered into the database for storage. This dataset is further filtered depending on the scientific question and cohort selection and downloaded for offline processing. The offline dataset is used to evaluate the cellular structure, genomic profiling, quantitative image analysis, and quantitative EEG analysis. Single modality studies or multimodal integrative studies are performed depending on the scientific question. The processed data is then uploaded back to the platform for future use.

To date, there has not been a comprehensive system that collects all the raw data and expands the scope of the system to accommodate datasets generated through basic, clinical, and translational research. Given the wealth of data and tissues produced during the clinical workup for epilepsy surgery, we have developed a system that collects clinical epilepsy data and spatially registers all EEG data onto brain imaging, allowing for precise spatial mapping of resected tissue samples at electrode positions (Figure 1). This data platform we call ‘INTUTION’ has enabled multimodal research studies on the tissues and integrated datasets, to develop a better understanding of the underlying disease and drive better treatment plans (2, 11, 12).

## Methods

INTUITION enables interdisciplinary collaborative studies. Along with the raw datasets from several clinical systems, INTUITION supports asynchronous data processing tools that help to de-identify EEG and brain imaging, post-process EEG for epileptic spike-seizure detection, create 3D brain models from imaging data, and facilitate genomics analysis. The system enables semi-automatic electrode co-registration of EEG electrode positions on 3D brain models and registers resected brain tissue samples to the 3D surface. INTUITION also supports inventory management for omics samples and maintains the scanned histology images at different scanning resolutions. Such a well-curated and controlled data set not only helps in surgical planning but also helps researchers to understand and develop new treatments for epilepsy (Figure 1). Here we describe our integrated informatics approach enabled by INTUITION that orchestrates several data pipelines to optimize clinical decision making in epilepsy and supports translational research.

## Patient population

INTUITION manages multimodal information from over two hundred epilepsy patients who have undergone two-stage epilepsy surgery in the past 20 years. Following patient informed consent, INTUITION initially collects all raw data, including identifiable features. Data de-identification modules remove all identifiable features when the data is moved from the clinical systems to the research environment. While all the patients had surgery for epilepsy, many of the patients had additional conditions, including polymicrogyria, tuberous sclerosis, focal cortical dysplasia, hippocampal sclerosis, brain injuries, and tumors, further enhancing the value of the tissue/data collection. We further link this patient data to a tissue bank containing over one thousand pieces of human brain tissues precisely mapped to each recording electrode. The patient population ranges from pediatric cases (as early as six months old) to older adults (above 50 years). With ongoing data collection and plans to use the platform at multiple sites, the platform aims to collect richer datasets from an even more diverse patient population.

## Patient data collection

At the University of Illinois at Chicago, the clinical care of epilepsy patients generates a significant amount of data, a copy of which is stored in the clinical research data warehouse (CRDW). INTUITION has an established data request with the CRDW to prospectively extract new epilepsy patient data to a REDCap project dedicated to INTUITION. INTUITON, through its automated data pipelines, downloads, transforms, de-identifies, and loads patient data to the database. Research users download the radiology images manually through a local PACS reviewer workstation and EEG data from the EEG lab in the hospital. The data details are as below.

### Clinical information

We populate de-identified clinical information, including demographics, clinical diagnoses, procedures, unstructured clinical notes (blobs), and reports of imaging and neuropsychological evaluation, into the system through REDCap APIs. Research coordinators manually enter the surgical outcomes, seizure observations, intraoperative surgery data, and copies of the original report files. The research team and physician always validate the information. Based on the different study designs and needs, we expand the scope of clinical information with additional information.

### EEG datasets

Every patient in INTUITION had scalp EEG recordings and the implantation of intracranial electrodes at precise brain regions using long-term video-EEG recording sessions. While storing the raw EEG data sets, the data size can expand to approximately 20 GB for one-day high-density EEG recording (124 channel, 1000 Hz sampling rate). If the data includes video files, it can expand to 40–60 GB per patient daily for video-EEG. During the entire recording period, this data can be up to several terabytes (TBs) (Table 1). For each patient, at least 3 × 10 min of EEG segments are collected from the intracranial EEG, which includes interictal activity. Location and EEG of seizures are also collected. The data is always reviewed and extracted under physician supervision to maintain the data quality. For each patient, we use a variety of algorithms to measure interictal epileptic waveforms and seizures and link these to precise locations using offline processing tools as described below.

**Table 1:** Data elements with approximate data size per patient.

Data domain	Data module	Data type	Data records	Data records
Clinical	Demographics	tabular (numeric/string)	1	KB
	Diagnosis	tabular(string)	∼10–100s	KB
	Procedures	tabular(string)	∼10–100s	KB
	Medications	tabular(numeric/string)	∼100s	KB
	Labs	tabular(numeric/string)	∼1000s	KB
	Notes	tabular(text)	∼100s	MB
	Ontology	tabular(string)	1	KB
Neuropsychology	All tests	JSON	∼1–2	KB
Outcome	Outcome	tabular(string)	1	Bytes
EEG	Reports	tabular(text)	∼1–10s	MB
	EEG files + meta info	file (.eeg/.edf)** **+** **tabular(string)	∼3–10s	1 GB—2TBs
Radiology	Reports	tabular(text)	∼1–30s	MB
	Imaging Files + meta info	file(DICOM RAW, NIFTI, DCM) + tabular(string)	∼1–30s	∼GBs
	3D electrode coregistration	file(DFS, OBJ) + tabular(string,C SV)	∼1–3s	<1GB
Surgery	Surgical Data	(JPG) + tabular	∼10s	∼100MB
Tissue	Images andinventory	(JPG) + tabular	∼1–10s	∼100MB
Histology	Images	(jpeg2000/TIFF) + tabular	∼1–10s	∼GB—TBs
Studies/Omics	Tissue usage & results	Files, hyperlinks	∼1–10000s	∼TB—PB

### Multimodal imaging

Imaging modalities are critical for localizing the seizure foci and related lesions. When combined with the intracranial EEG studies, they provide the spatial framework for designing therapeutic brain resections. Brain imaging includes multiple magnetic resonance imaging (MRI) sequences, computed tomography, x-Rays, positron emission tomography, single photon emission ictal scans, magnetoencephalography, and event-related optical imaging. While not all the recordings or scans are performed on each patient, usually one or more tests and scans are performed based on the need. For each patient, pre-electrode implantation, post-electrode implantation and post-operative imaging scans are collected, which are needed for the co-registration of EEG electrodes on a multitude of imaging studies. INTUITION de-identifies and stores raw images used to understand brain structure, volumetric information, structural connectivity, tissue co-registration, identify brain structures, highlight abnormalities and perform computational image analysis (13–15).

### Tissue samples

Tissues removed as part of the surgical procedure and not needed for diagnosis are used for research purposes and stored in our NeuroRepository following informed consent. These samples post-surgery is immediately mapped to the precise location of the brain using pictures in the surgery room and brain arterial patterns. Further, these samples are co-registered with EEG and MRI following standardized protocol (2). These stored samples are used for histological analysis, staining, genomics, proteomics, and metabolomics. In this way, each brain location with specific imaging features and corresponding electrical properties recorded from *in vivo* (e.g., spikes or seizures) can be linked to tissue histology and molecular/genetic attributes. INTUITION provides an inventory of the entire dataset, including tissue stored in refrigerators, sectioned tissue slides, RNA, DNA, and protein inventory (quantity remaining and storage location), and the links to EEG electrodes, EEG quantified results (spikes, seizure onset), MRI co-ordinates (for comparison with brain lesion locations). This information is used for cohort selection and research.

## System design, infrastructure and management

**Figure 2:**
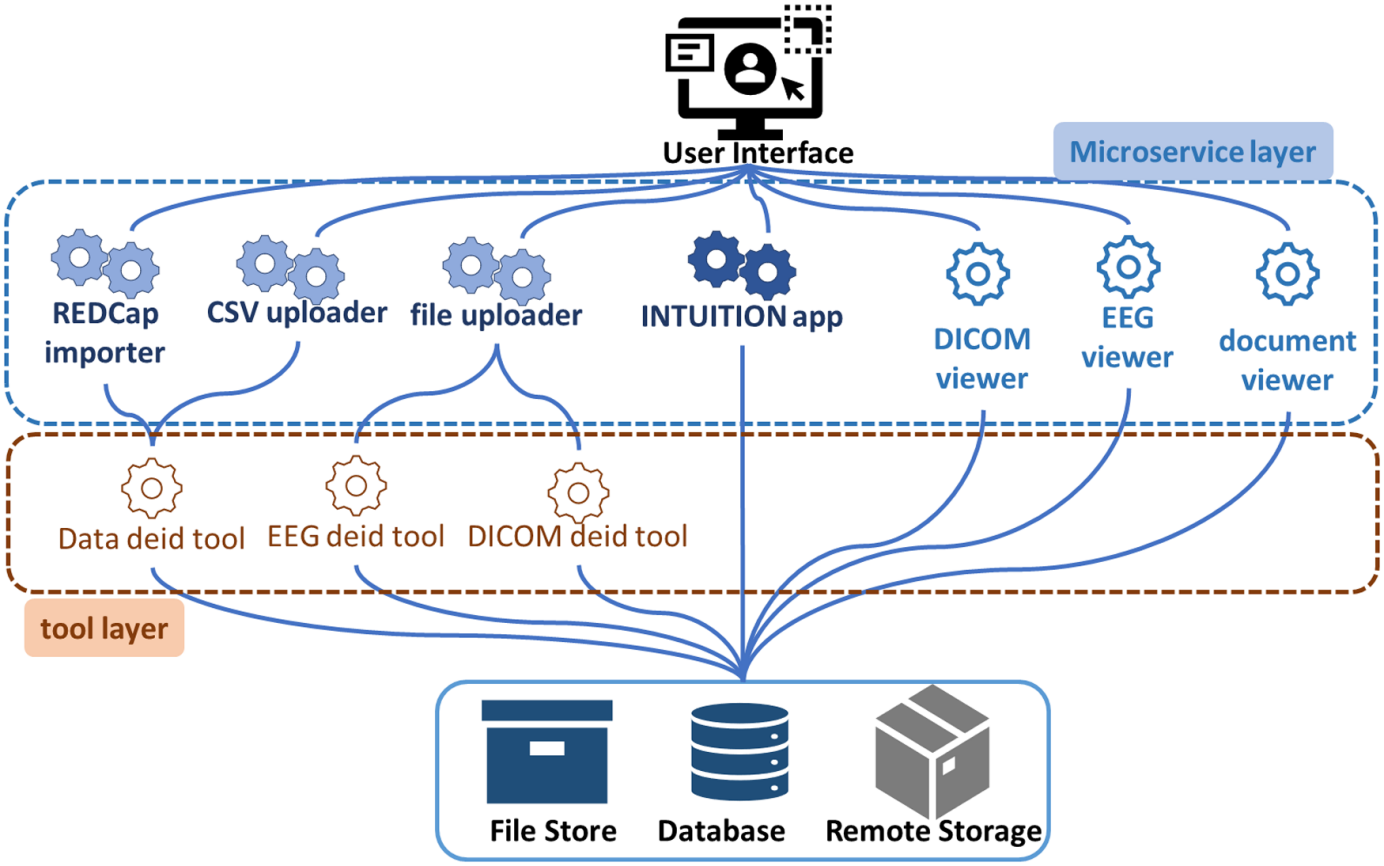
INTUITION system design.

INTUITION is developed on Django's model-view-template (MVT) architecture with PostgreSQL database, Python middleware, Django web framework, JINJA template engine, and HTML, CSS, and JavaScript for additional frontend work. An outline of the system architecture is shown in Figure 2. The system has a base application along with several service apps to support tools, viewers, and data operation (Figure 2). Some packages used for the application are Django Object Relationship Mapper (ORM) to interface with the database, Django-Migrate for database migrations, Django-REST for building RESTful APIs, Django-Cryptography for hashing utilities, Django-SimpleJWT to enable JSON Web Tokens (JWT) for API and other security features, Django-CORS for handling Cross Origin Resource Sharing (CORS), Allauth for handling authentication and authorization. We used the Requests library for making HTTP requests, Pandas for data transformations, Plotly for visualization, and CRISPY Forms for creating forms in the templates, which provides advantages of implementing bootstrap v5 forms while enforcing form functionalities like marking fields as required, hidden, dropdowns and multi-select checkbox. We secure the forms from cross-site request forgery using Django inbuilt functionality of Django CSRF, handling file uploads and JSONminify to reduce data transmission load between the client and server. We use the inbuilt Django-Storage library in the backend to create the file upload field and data storage management within the media files, which improves the efficiency of reading and writing files compared to using file storage in a database. We used Jinja2 templates, HTML (Bootstrap 5), and JavaScript to make the user interface seamless. We have followed Jakob Nielsen's ten general heuristics for interaction design to ensure our user interactions are seamless and on par with the global UI/UX standards (16). We also have an asset caching system that allows us to reduce latency between request and response by caching static resources in the user's browser. We also included features to bulk import and export CSV data from the system. We also use the PyDICOM library to process DICOM files and manipulate them to reduce payload for transmitting it to the client and pylibjpeg to manage image modifications and functionalities like contrast, brightness, and rotate images. We use the Django Simple History library to log all the data creation and modifications performed on Intuition. The Django-Authentication module manages the user access control. The library manages user authentication, session management, password hashing, password validation, and handling of password reset requests.

We used PostgreSQL (v13) as our database because it is robust for online transaction processing (OLTP) and a competent analytical database that integrates well with Django. PostgreSQL also can manage high volumes of scientific data, is scalable, can be easily extended, and is freely available. The files for EEG, imaging, and histology module, along with any document, were in the Windows Encrypting File System (EFS); the index of these files was stored in a relational table with entity-attribute-value (EAV) data model. In the case of the EEG and Imaging dataset, where the uploaded file count for each patient is quite variable, and each entry can have associated support files (specifically for EEG), we have used a simple JSON field in corresponding tables to store names and associated files with file tags, file types, extensions, and associated user-created files.

The system's interactive search lets users search through any information available on INTUITION. The basic search enables users to enter keyword-based search terms parsed by the system to display a list of available patients. Additionally, an advanced search lets users select predefined search boxes for more complex patient selections.

INTUITION has a concept of PROJECT, which aggregates a set of patients for a particular research study and users who have access to that project enabling investigators to create study cohorts within the platform and share it with collaborators.

INTUITION is hosted on a Windows server (IIS version 10.0 on Windows 2022). INTUITION maintains end-to-end TLS using HTTPS protocol. Currently, the INTUITION application is deployed within the University of Illinois Chicago Private network for an added layer of security.

## Offline data processing in INTUITION

### Data de-identification

We have developed a set tool to de-identify each patient’s imaging and EEG data.

**DICOM de-identification:** We have developed a web application tool that uses Pydicom to perform image modifications. The program works in two steps. First, it reads all the attributes of the DICOM header of the uploaded series. It deletes the header elements that are private and unknown attributes (VR or UN tag) as per the DICOM standard. It uses three sets of predefined attributes to perform de-identification. The patient name is updated with user-entered shifted data and patient code for the attributes marked as “Update Fields,” such as study date. It will assign random string values to these attributes if these fields are not provided. Attributes defined as “Keep Fields” are kept as it is. These attributes are key to understanding the scan details, parameters, data orientation, and the data matrix. The “Remove Fields” attributes must be deleted if found. These fields correspond to accession numbers, machine serial numbers, hospital addresses, and provider details. In the second step, the tool displays the DICOM pixel data to the user to select regions needing removal from the images. The users can select one or more rectangular regions and whether they need to be deleted from one slice or all slices of the DICOM series. Once the selection is made, the tool removes the pixel values and regenerates the DICOM images in a de-identified format. Further, the data can be stored on INTUITION, or the user can download it to their local environment.

**EEG de-identification**: The EEG is always extracted in European Data Format (EDF). The EEG de-identification tool reads the EEG file headers, removes, or modifies the fields that correspond to name and data attributes and rewrites the data back to EEG file in a fashion like the image de-identification. Further, we also remove all annotations from the file and either store it on INTUITION or let the user download them.

**OMICS data de-identification:** OMICS data de-identification is a complex process that involves ethical, legal, and technical challenges. To de-identify OMICS data, we follow several steps that involve technical, data governance, and sharing policy changes. The details of these steps are as follows. First, we remove personally identifiable information from the standardized omics files, such as patient names, IDs, age, gender, clinical conditions, and tissue location. Second, we manage the coded OMICS data on a separate server with limited connectivity to the INTUITION system. Access to this server requires additional privileges. Third, INTUITION holds derived information from genomic data, which has a minimal scope of re-identification. The genomic store is also encrypted to ensure added security. Fourth, complete genome records are never shared without appropriate request and approval from the data governance committee.

Fifth, data access is limited to requests with valid scientific purposes, and minimum data is shared based on the type and scope of the data request. Finally, upon a suitable agreement, we share an aggregated and coded dataset. The process of re-coding and aggregation reduces the risk of patient re-identification.

Overall, these steps aim to ensure that OMICS data is de-identified and shared in a secure and responsible manner.

### EEG and MRI analysis

Our primary goal is to analyze EEG data to identify epileptic events such as interictal spikes and epileptic seizures, which are the electrophysiological biomarkers of epilepsy; co-register these event's occurrence, location, and propagation patterns (13, 17) across brain locations over 100 electrodes on a 3D brain model. Once the tissue is resected, perform additional co-registration of their locations on the same 3D model. In addition, we can examine many other EEG features from the raw data not limited to periodic discharges, frequency band-specific epilepsy signatures, and high-frequency oscillations. The computational work is performed offline using in-house algorithms and scripts developed in MATLAB and Python, and the processed information is passed back to INTUITION. While the initial design used MATLAB as the offline tool for signal processing, the data can be connected to other analytical platforms through appropriate APIs. In relation to the EEG work, we have developed an EEG de-identification tool, an EEG visualization tool.

For brain images, we use established tools like BrainSuite or *freesurfer* (18) for offline 3D re-constructions of the brain, cortical thickness, and geodesic distance measures. We use an in-house developed algorithm to use MRI and CT to co-register the electrode location and match it with intra-operative images for accuracy. This method provides an accurate localization of the electrodes and corresponding geodesic distance and cortical thickness. Furthermore, since many patients with epilepsy have brain lesions such as developmental abnormalities or tumors, our multimodal analytical framework also localizes these lesions in relation to the electrode locations. This helps us understand lesional and non-lesional epilepsy in a detailed manner. To manage brain imaging data, we have a DICOM-de-identification tool, DICOM visualization tool, and 3D model rendering tool that are web-based and integrated into INTUITION.

### Histological and molecular/-omics analysis and image data storage

A unique characteristic of our approach is that each piece of resected brain tissue is precisely mapped to a specific brain electrode location. This enables a direct link between brain structure and electrophysiology at that specific region to the histology (or cell structure) and molecular features of the underlying tissue. Our previous work outlines the meticulous way we subdivide each block of tissue underlying a specific brain region for these analyses. High-resolution digital images of stained sections from each brain region, genomics (19–21), proteomics (21), and metabolomic (22) are stored within INTUITION and readily available for focused, discovery-based projects to understand and develop better treatments for epilepsy. While INTUITION stores the results of omics analysis on-premises, the raw files, often large (Table 1), are stored on the data server, and their links are stored on the INTUITION inventory module.

## Results

**Figure 3:**
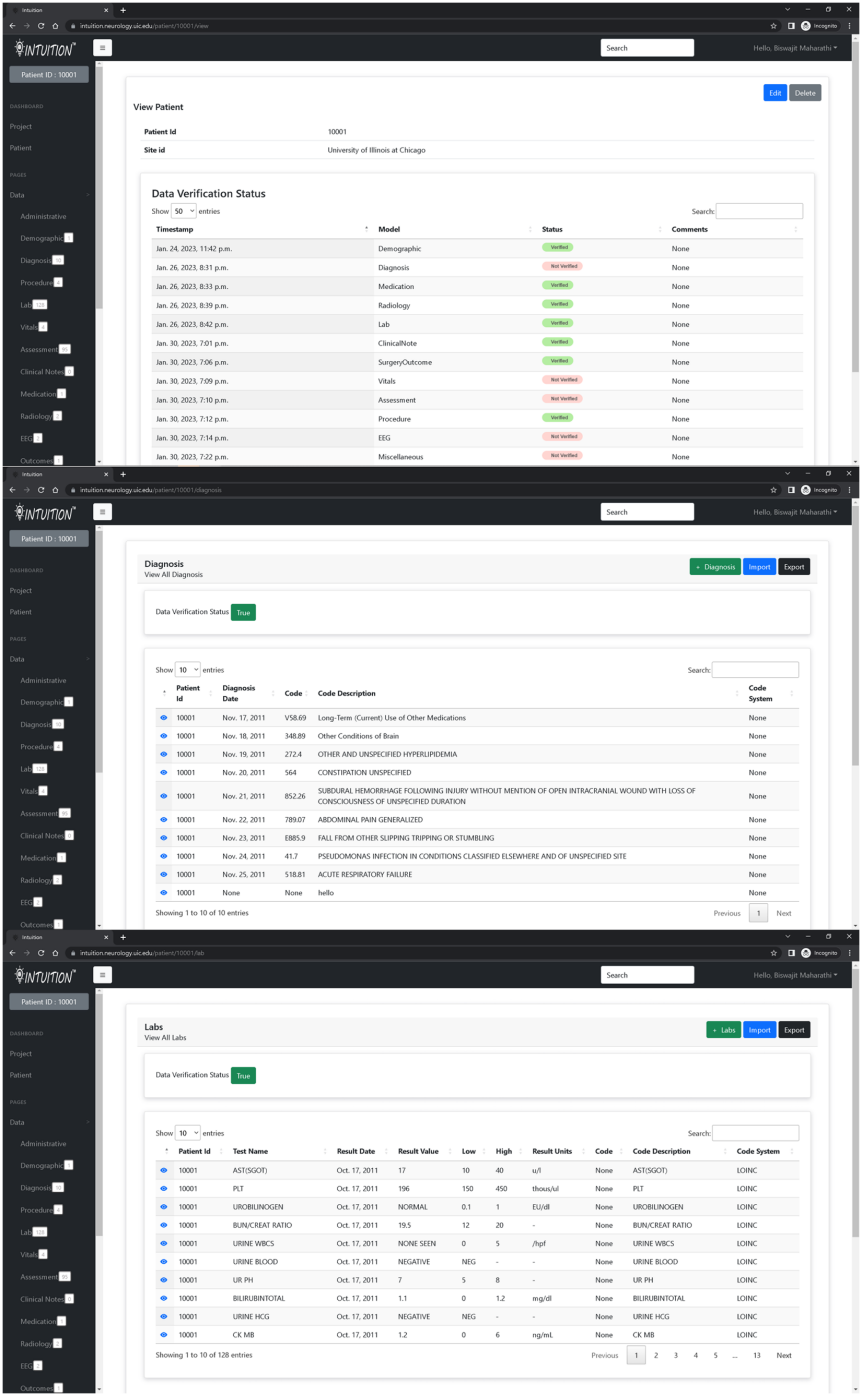
INTUITION pages displaying information extracted from electronic health records. The top figure shows the overall status of all modules whereas the sidebar status shows the records available within each data module. The middle and lower image shows sample of diagnosis and lab measurements for patient 10001.

The INTUITION application currently holds two hundred epilepsy patient data totaling 2 TB of on-premises storage and several terabytes of omics and imaging data on remote servers. The system supports a status dashboard that displays the status of data entries and whether a data reviewer has verified them. The navigation sidebar additionally provides information regarding the record counts for investigators to know how much data has been collected for each patient (Figure 3).

**Figure 4:**
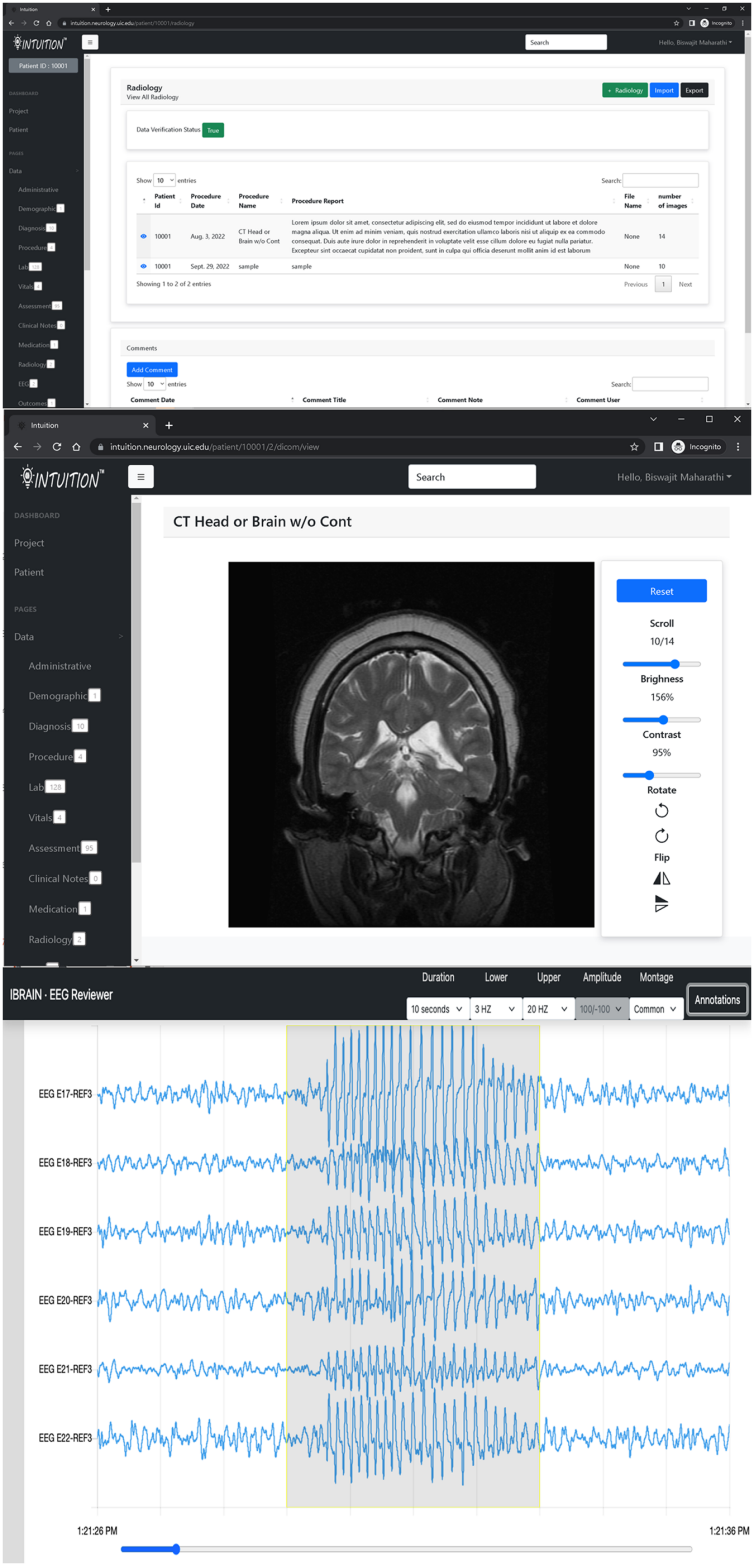
INTUITION imaging and EEG visualization tool. Top two panels display an MRI record with capability to review radiology notes and the DICOM image series on an inbuilt browser. The bottom panel shows the EEG browser with sample EEG data with a seizure event annotated.

In addition, INTUITION has a patient timeline and data dashboard view (Figure 4) that has been tested for a previously published expansion on traumatic brain injury and epilepsy (10).

## Search and cohort discovery

INTUITION provides a simple search interface that accepts text-based search criteria for demographics, diagnosis, and medications; and returns the qualifying list of patients. This list can be further aggregated, stored as a project, and can be shared with other investigators or reused at a later point. INTUITON also provides advanced search features that help in cohort discovery. The advanced search page takes information such as demographic filter (age, gender, date ranges), presence of any diagnosis code (ICD codes or descriptive text), prescribed medication, procedures conducted, presence of specific lab measurements, and the range of values for that measurement, availability of the specific type of medical imaging scan along with the number of times the scans was performed (e.g., Pre and post-surgery MRI scans), availability of EEG, tissue inventory, and keyword-based search through available unstructured clinical notes.

## Challenges ahead

We have created a multimodal database platform for uploading, storing, searching, and analyzing system biology datasets collected from two-stage epilepsy brain surgery patients. The platform INTUITION, named to mimic what a physician requires to make a diagnosis after absorbing many types of data, currently holds two hundred well-curated patient datasets along with location co-registered tissue samples used for histology and omics analysis of thousands of brain regions. While this form of ‘Big Data’ is not large compared to other datasets, the comprehensive, multimodal nature allows discoveries not possible with other, larger, but less integrated datasets. This is achieved through direct linkages between clinical, electrical, imaging, histological, and molecular data. Having both a clinical and research environment protects the patient's identity and enables the connection of all deidentified data modalities for the same patient. INTUITION and the datasets within INTUITION have led to important discoveries summarized in a recent review article (2). These discoveries range from improving our fundamental understanding of epilepsy to new therapeutics, new diagnostic approaches, and discoveries about what makes the human brain unique. The INTUITION system has fostered several patent applications, new drugs, and brain imaging strategies that would not have been possible without a highly curated, integrated, multimodal dataset of the human brain.

Moving forward, this platform has multiple challenges that are currently being addressed through further development of the INTUITION platform: (1) Finding ways to integrate the heterogeneous datasets for each patient and across all patients at multiple sites/surgical programs to create a secure, federated platform of deidentified data; (2) The search for epileptogenic biomarkers presents specific challenges on combined electrophysiology and imaging data and histology-omics data requiring new, offline workflow processes to create metadata sets that reduce the complexity for meaningful insight; (3) The platform is highly labor-intensive and requires a significant amount of manual data processing and entry; (4) Most datasets that feed into INTUITION reside on different, siloed servers that do not link to one another.

## Conclusion

INTUITION is a unique data platform that brings together multiple data types within a focused human disease. Building this for patients who undergo epilepsy surgery provides some of the most detailed multimodal data that exists on the human brain linked to fresh human brain tissue samples. While not a large database, the carefully curated metadata for each patient offers an unparalleled opportunity to understand and develop novel diagnostic and treatment approaches for patients with epilepsy. Advances made in the building, expansion, and automation of INTUITION will further advance its utility and allow discoveries in many other human disorders.

## Data Availability

The original contributions presented in the study are included in the article, further inquiries can be directed to the corresponding author/s.
